# Current News

**Published:** 1890-12

**Authors:** 


### Current News.

A METHOD of adding gum to ordinary artificial teeth. By George Cunningham, M.D. (Cantab.), D.M.D., D.D.S., R.C.S. (Eng.).

A reference to the formulæ for continuous-gum work in that very interesting chapter on moulding and carving porcelain teeth in the "American System of Dental Surgery," vol. ii., shows that they consist of ingredients of very different degrees of fusibility, and it seems to me that such ingredients as cryolite, Bohemian glass, flint glass, and "white glass" (whatever that may mean) are added for the purpose of reducing the fusibility or acting as a cement to the more refractory ingredients, such as silica or quartz, kaolin, and spar. I therefore set to work and instituted a series of experiments which may be briefly described as the very opposite; that is, adding the more refractory substances, which for convenience of discussion may be termed "tooth frit," for the purpose of giving stamina and cohesion to glass as a basis. Having satisfied myself as to the possibility of making an artistic and natural reproduction of gum color with mixtures of ordinary colored glass and vitreous enamels of various kinds, and also the possibility of controlling the fluidity, if I may be allowed the expression, of the molten glass, I found from a consultation of technical literature on the subject, which is wofully unsatisfactory from a purely scientific point of view, that, as we would anticipate, there is a very considerable difference as to the fusibility and the solubility of the various kinds of glass. After various and prolonged experiments which it would be tedious to detail, suffice it to say that I succeeded in turning out in this way an artificial denture of enamelled platinum which enabled me to approach one of the highest technical authorities,—namely, Mr. Harry Powell, of the celebrated White Friars Glass-Works, London,—with a view to interesting him in my experiments. . . . He very kindly placed his practical knowledge and technical skill at my disposal. By utilizing a formula for that mosaic work for which this ancient house is famous, we have succeeded in producing a body and enamel capable of fusing at a relatively low temperature. With regard to the artistic results achieved, the specimens which I now exhibit speak for themselves, even though the experiments are not yet complete. . . .

After having referred to the extreme fusibility of this new enamel, you will doubtless be surprised that the specimens presented to you are mounted on a highly-infusible metallic base. The new materials may be fused on copper, dental alloy, and gold, but it was early discovered that there was so far only one material—namely, platinum—which was available, and that for two reasons. First, during heating, chemical change takes place between one or more ingredients of the enamel and the metallic base, such as eighteen-carat gold, which has hitherto prevented my obtaining the natural gum colors on any other dental metallic base but platinum and pure gold. This change might possibly be obviated by using a glass which did not contain silicate of lead, but as there are other qualities to be considered, such as durability, strength, and solubility, it is considered that we obtain a stronger material, insoluble, or at any rate practically insoluble, in the mouth by keeping to the use of flint glass as the main ingredient.

Recent experiments on the behavior of this vitreous enamel on various metallic bases afford a reasonable clue as to the cause of the change of color in the vitreous enamel. Some of my specimens prove,—

1. That on pure gold there is no discoloration.
2. That on silver there is a yellow discoloration.
3. That on copper there is a black or greenish discoloration.

These facts seem to indicate, first, an oxidizing of the metal under the influence of heat, and, secondly, the metallic oxide, thus formed, imparting its color to the vitreous enamel, either directly or by causing some further chemical change in the constituents of the vitreous enamel. A similar discoloration takes place with the alloys, and therefore, in eighteen-carat gold, we obtain so much discoloration, both from the copper and the silver it contains, as to preclude its use in this method. So readily is the vitreous enamel discolored, that even on the pure gold specimen the one or two tiny points where coin gold was used as a solder, or to close a small fissure in the plate, were distinctly marked by a deep green local discoloration on baking the body.

Secondly, the coefficient of expansion of platinum and glass being the same, platinum must possess practically obvious advantages, especially as to adhesion, over any other material. If it be desired to give the denture the more acceptable appearance of gold, it is easy of accomplishment, as, for instance, by electro-gilding. Another method, which is not uninteresting, is well represented by this speci[illegible]. The metal base is made by sweating a piece of pure gold and pure platinum together and rolling them out in the mills to the desired gauge, the enamel is infused upon the platinum surface, leaving an exposed surface of pure gold. Mr. Powell was extremely surprised to see the adhesion of the vitreous enamel on pure gold; although the attachment is not so strong as in the case of platinum, it is evident that by stippling we can get sufficient attachment for our purpose, though this statement may have to be revised later on, as no practical case on gold has yet been worn in the mouth for any length of time. On dental alloy, as one of the specimens shows, the enamel simply flakes off as the specimens cool from the unequal contraction of the dental and the enamel. It is still necessary by this method to use pure gold for the soldering of the teeth to the platinum base; but soldering, however, is not absolutely necessary, as demonstrated by this full set of continuous gum. Here the teeth were mounted as usual in wax on the metal plate; the case was then set teeth downward on a base of plaster, sand, and fire-clay, equal parts, the investment being carried over so as to embrace the tips of the teeth, and thus to hold them in position. The wax was then removed in the usual way, and the body built up around the teeth; the purple color which shows behind the front teeth in this specimen is due to a chemical action between the investment and the body, and indicates a danger, which, however, can usually be avoided. Although the enamel seems to adhere with tolerable firmness to the smooth platinum plate, it is better to increase its attachment by either stippling the plate or forming a boundary for the material by means either of a turned-up edge to the plate, or, what I think is better, soldering a rim of triangular wire of platinum with pure gold; all of which processes are exemplified in this specimen case (full upper). In this same case you will see that the enamel is equally applicable to English and American teeth, the front teeth being American, and the bicuspids and molars "Ash's diatoric."

For full, but especially for partial, dentures, both upper and lower, this new enamel seems to afford a great and important sphere of usefulness for the excellent English tube work. One reason why this work is so little employed is no doubt due to the fact that too frequently the dental mechanic of to-day is lacking either in the ability or in the patience requisite in nicely and accurately adjusting the tube teeth to the plate. This fine fitting of tube teeth, which occupies, even in the hands of an expert, the greater part of the time of manufacture, is entirely obviated by the new method of working it. The plate is struck up in platinum, and, instead of gold, platinum pins are mounted in the usual way, only soldered with pure gold. No fine fitting of the teeth to the plate is necessary, as the body does that more effectually than the most expert manipulator of the corundum wheel. The use of sulphur cement and the working loose of the teeth is also obviated, since they are held firmly in position by the body of the enamel. The general excellence of ordinary tube work is further improved by the filling up of all spaces where food might lodge, while, without impairing in any way the utility and strength of the other method, the artistic coloring of the restored gum is, I think, a great advance on the often unsightly long-rooted tube teeth. . . .

For small blocks of a few teeth, I think it is evident from these specimens that the new method is of considerable utility and of artistic value in special cases. For small cases of bridge-work, removable or fixed, this enamel seems to have a great field of usefulness.

With regard to the process of firing, I have had great difficulties, as no existing form of furnace was found exactly applicable to the dimensions of the ordinary denture. If, however, the profession adopt this method of continuous-gum work, such difficulties will be easily overcome, as, by constructing and adapting a platinum muffle to the ordinary small Fletcher's muffle furnace, I have been enabled to turn out the specimens which have been exhibited to you. The furnace is simply an ordinary draught gas and air furnace, and the whole process of firing can be accomplished in about a quarter of an hour, though it is sometimes advisable to take a little longer time.—*Condensed from the Journal of the British Dental Association.*

---

We learn that the *Archives of Dentistry* will cease to exist after this year. This is a matter of sincere regret, as the *Archives* has been one of our most valuable journals.

---

Dr L. P. Haskell says this of Babbitt's metal in casting:

"The dentist who says he has found 'no metal so reliable as zinc' has never used a *proper* Babbitt. It is not the *pouring* of zinc that alone gives trouble. It necessitates the use of *wet* sand, and the constant necessity of guarding against air-bubbles, a trouble that never occurs with oiled sand, which can be packed hard and is always ready for use. And the man does not live who can fit plates as easily and as uniformly perfect with zinc as with Babbitt.—*Ohio Journal of Dental Science.*

